# Assessment of pathogenicity and tissue distribution of infectious bronchitis virus strains (Italy 02 genotype) isolated from moroccan broiler chickens

**DOI:** 10.1186/s12917-016-0711-y

**Published:** 2016-06-08

**Authors:** Khadija Khataby, Faouzi Kichou, Chafiqa Loutfi, My Mustapha Ennaji

**Affiliations:** Laboratory of Virology, Microbiology, Quality and Biotechnologies/Ecotoxicology & Biodiversity, Faculty of Sciences and Techniques, University Hassan II of Casablanca, Mohammedia, Morocco; Society Biopharma, Km 2, Route de Casa, B.P. 4569, Rabat, Morocco; Department of Veterinary Pathology and Public Health, Hassan 2nd Institute of Agronomy and Veterinary Medicine, B.P. 6202, Madinat Al Irfane, Rabat, Morocco

**Keywords:** Infectious bronchitis virus (IBV), Italy 02, Clinical scoring, Pathogenecity

## Abstract

**Background:**

Avian infectious bronchitis (IB) is one of the most important viral diseases of poultry, affecting chickens of all ages and causing major economic losses in poultry flocks. Mass vaccination is conducted in Morocco using a vaccine against Massachusetts, which is the most dominant serotype; however no information about the pathogenesis and tissue distribution of the Moroccan Italy 02 genotype was reported.

40 one-day-old specific pathogen free chickens were divided randomly into four groups. Group1, 2 and 3 were inoculated intra oculo-nasally with 103.5 EID50 of Italy02 viruses, and group 4 was kept as control. Chickens in each group were monitored for 14 days post-infection (pi).

**Results:**

Chickens in all infected groups showed severe respiratory signs, which most of them have been reproduced on 2dpi, with varying times of appearance and disappearance. The infected birds appeared lethargic, reluctant to move, with specific respiratory clinical signs and macroscopic lesions. However no nephritis lesions or mortality were recorded in all groups. The specific histological lesions finding in all infected birds, exhibited tracheal lesions with mucosal thickening, hyperplasia of the surface epithelium, mononuclear inflammatory cell infiltrate of lamina propria. Primary and secondary bronchi, epithelial hyperplasia and mononuclear inflammatory cell infiltrate of the lamina propria were also observed. Tracheal lesions developed in all infected birds, confirm the ability of the three tested strains to induce respiratory disease. The results at 14 dpi also revealed that all strains were able to induce serological response. Virus re-isolation from infected organs and amplification of the viral RNA by real-time PCR proved the presence of the virus in lung and trachea of infected chicks. Neither re-isolation nor significant viral RNA detection were detected in the kidney.

**Conclusion:**

The results demonstrated that the three strains Italy02 genotype emerging in Moroccan poultry farms have a wide distribution for respiratory system, without kidney damage and without causing mortality.

## Background

Avian infectious bronchitis (IB) is an acute, highly contagious respiratory disease of chickens, causes major economic losses in poultry industry worldwide [1]. The IB virus (IBV) is a member of *Gammacoronavirus* genus, previously Group 3, within the Coronaviridae and it is the type species of the avian *Coronavirus* of the domestic chicken (*Gallus Gallus*)) [2].

It is generally accepted that chickens are the most important natural host of IBV and epithelial cells of the upper respiratory tract are the primary target, and intensive virus replication, predominantly in the trachea, results in respiratory signs, which are the most frequent clinical manifestation of this disease [1].

Chickens of all ages are susceptible, but the severity is great in younger ages [3], and the clinical signs include depression, coughing, dyspnea, sneezing, nasal discharge, and death [4]. However, some strains of IBV can also replicates in the ciliated epithelial cells of organs, such as the kidney, reproductive and enteric tracts, producing severe nephritis, reproductive disorders in males and females, a drop in egg production and quality in laying flocks and deep pectoral myopathy in broiler breeder may occure [5].

The transmission of IBV is mainly horizontal by direct contact via the respiratory tract from infected chickens. Infection takes place via inhalation of droplets containing the air born virus. Or indirect by contaminated feed and drinking water, including human beings, probably contribute to more local spread [6]. In addition, it has been demonstrate that certain strains of IBV may persist in small amounts in the cecal tonsils of the intestinal tract by asymptomatic way during long time [7].

IBV is an enveloped, non segmented, positive sense, single stranded RNA virus. Its genome consists of about 27.6 kb and codes for four structural proteins: the membrane (M), small membrane (E), nucleoprotein (N) and spike (S) [8]. The multimeric coiled-coil S protein is post-translationally cleaved into smaller proteins namely S1 and S2 [8]. The S1 gene contains the hypervariable regions that are responsible for the induction of neutralizing, serotype specific antibodies and protective immunity [9]. Many IBV genotypes and serotypes have been identified and have complicated efforts at control through vaccination, due to the frequent point mutations in S1 gene that can be partially or poorly neutralised by existing vaccine serotypes [10]. For this reason, the sequencing of this gene is the most useful strategy for the molecular characterization of virus isolates existing in the field and the selection of appropriate vaccines [11].

In Morocco, IBV was identified for the first time in 1983 by El Houadfi & Jones [12]. Subsequently, several reports confirmed the IBV strains related to the Massachusetts and to 4/91 genotypes [13, 14].

Recently, between 2010-2014, an epidemiological survey showed the emergence of a novel strain of Italy02 serotype with a prevalence of 32 %, co-circulating with two serotypes; Massachusetts and 4/91, with a prevalence of 66 % and 2 % respectively, that are isolated from vaccinated and unvaccinated chicken flocks [15].

Mass vaccination in Morocco is conducted using a vaccine against Massachusetts, which is the most dominant serotype, however no information about pathogenesis and tissue distribution of Italy 02 serotype, hence the objective of this present study which is reported for the first time in Morocco, aims to evaluate the pathogenicity and the tissue distribution of the three isolated Moroccan strains of IBV Italy 02 genotype in one day old experimentally infected SPF chickens. The clinical signs, tracheal ciliary activity, gross and microscopic lesions were evaluated. Serological response by the detection of IBV antibodies of the affected chicks was also checked. Re-isolation of the virus from the affected organs and RT-PCR test was used to detect virus in several tissues of infected birds.

## Results

### Clinical signs and gross pathological findings

Clinical monitoring of infected chicks reveals at first, apparent respiratory symptoms beginning at day 2 post-inoculation (dpi). Respiratory clinical signs were predominant in all of the inoculated groups and were intense and more severe until 7 dpi, with no clear differences in the pathogenicity of the three strains. The most prominent clinical signs were characterized by gasping, depression, sneezing, difficulty in breathing, cough, pulmonary and tracheal rales, with high scores were reported for all the tested strains with clinical score of 108, 126 and 140 for IBV/RA, IBV/MN and IBV/TU strain respectively, (Tables 1, 2 and 3).

**Table 1:** Clinical Signs scores of SPF chickens inoculated with IBV strains IBV/MN*IBV* infectious bronchitis virus

IBV/MN Strain						
Days Post inoculation	Clinical Score (0 to 3)					
	No Signs (0)	Sneezing (1)	Tracheal Rale (2)	Pulmonary Rale (2)	Sick (3)	Day Score
1	5/10	3/10	1/10	0/10	1/10	8
2	3/10	4/10	2/10	0/10	1/10	11
3	1/10	3/10	3/10	0/10	3/10	18
4	0/10	4/10	4/10	0/10	2/10	18
5	0/10	3/10	3/10	2/10	2/10	19
6	0/7	4/7	2/7	0/7	1/7	11
7	1/7	4/7	1/7	0/7	1/7	9
8	2/7	3/7	1/7	0/7	1/7	8
9	2/7	3/7	1/7	0/7	1/7	8
10	2/7	2/7	1/7	0/7	1/7	7
11	3/7	3/7	1/7	0/7	0/7	5
12	4/7	2/7	1/7	0/7	0/7	4
Total score	126					

*IBV* infectious bronchitis virus

*SPF* Specific pathogen free

*IBV/MN* infectious bronchitis virus sampled from Meknes Tafilalet region

**Table 2:** Clinical Signs scores of SPF chickens inoculated with IBV strains IBV/RA*IBV* infectious bronchitis virus

IBV/RA Strain						
Days Post inoculation	Clinical Score (0 to 3)					
	No sing (0)	Sneezing (1)	Tracheal Râle (2)	Pulmonary Rale (2)	Sick (3)	Day Score
1	9/10	1/10	0/10	0/10	0/10	1
2	3/10	4/10	2/10	1/10	0/10	10
3	2/10	3/10	2/10	2/10	1/10	12
4	1/10	2/10	4/10	2/10	1/10	18
5	0/10	3/10	6/10	1/10	1/10	14
6	0/7	3/7	2/7	2/7	0/7	11
7	1/7	4/7	2/7	0/7	0/7	8
8	1/7	4/7	2/7	0/7	0/7	8
9	2/7	3/7	2/7	0/7	0/7	7
10	2/7	2/7	3/7	0/7	0/7	8
11	3/7	1/7	2/7	0/7	0/7	5
12	3/7	2/7	2/7	0/7	0/7	6
Total score	108					

*IBV* infectious bronchitis virus

*SPF* Specific pathogen free

*IBV/RA* infectious bronchitis virus sampled from Rabat-Sale-Azzemour Zaire region

**Table 3:** Clinical Signs scores of SPF chickens inoculated with IBV strains IBV/TU*IBV* infectious bronchitis virus

IBV/TU Strain						
Days Post inoculation	Clinical Score (0 to 3)					
	No sing (0)	Sneezing (1)	Tracheal Râle (2)	Pulmonary Rale (2)	Sick (3)	Day Score
1	7/10	2/10	1/10	0/10	0/10	4
2	5/10	2/10	3/10	0/10	0/10	8
3	4/10	2/10	2/10	1/10	1/10	11
4	2/10	1/10	2/10	2/10	3/10	18
5	1/10	1/10	3/10	2/10	3/10	20
6	0/7	1/7	2/7	1/7	3/7	16
7	0/7	1/7	1/7	2/7	3/7	17
8	0/7	2/7	1/7	2/7	2/7	13
9	0/7	3/7	1/7	1/7	2/7	13
10	2/7	2/7	2/7	1/7	0/7	8
11	2/7	2/7	2/7	1/7	0/7	7
12	2/7	3/7	1/7	0/7	0/7	5
Total score	140					

*IBV* infectious bronchitis virus

*SPF* Specific pathogen free

*IBV/TU* infectious bronchitis virus sampled from Abda-Doukkala region

**Fig. 1:**
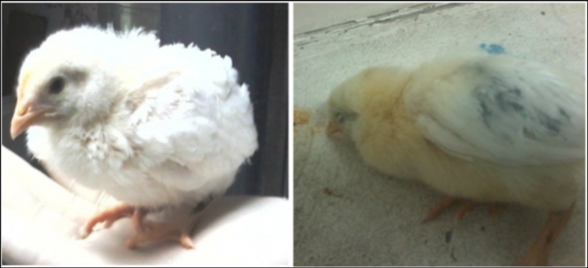
Birds lethargic and reluctant to move after infection with IBV/MN strain (in the left) and with IBV/TU strain (in the right)

Nasal discharge and watery eyes were also observed but were transient in some of infected by IBV/RA and IBV/MN chicks. These clinical symptoms were persisted in all groups until 12dpi. The birds infected by IBV/MN and IBV/TU appeared lethargic, reluctant to move (Fig. 1), whereas, chicks infected with by IBV/RA were not as apathetic. At autopsy, all infected chicks that were killed at 5 dpi to prevent their suffering were examined for the macroscopic lesions in the trachea, lung and kidney. These gross lesions consisted in hemorrhagic tracheitis, mucosal congestion and catarrhal exudates that mainly progressed. For the three strains used, the signs were observed in the most infected chicks with dominance in the lungs that were hemorrhagic and sometimes cyanotic at one lobe. Therefore, samples were taken at 14 dpi just for a macroscopic examination of organs to confirm whether the absence of clinical signs is correlative with the gross lesions. Whereas, gross lesions of kidney, were not observed in all inoculated chicks. During the experiment, the non-infected control group stayed normally without clinical signs or gross lesions. The statistical analysis was not performed as the three tested strains are phylogentically related to each other and the difference in clinical and tissues scores is not very significant.

### Histopathological examination

The microscopic tissues analysis was performed in the euthanized chicks at 5 dpi. This period correlates with the maximum intensity of clinical signs (which begins between 2 and 7dpi). These signs fade from the 7dpi with a total restoration at 12 dpi, that why we only conducted the macroscopic examination at this time. Histopathological lesions developed at 5dpi in the three groups of experimentally infected chickens, were compatible of those previously described for infectious bronchitis virus [16]. And their scoring was mainly correlating with the ranking and scoring described in previous experiments [17].

**Fig. 2:**
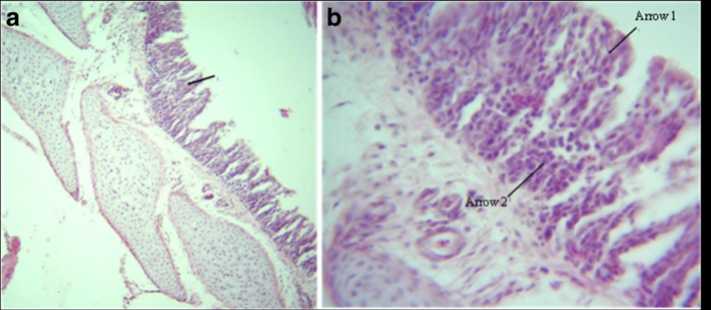
**a** Trachea of IBV/TU inoculated chick – Severe thickening of mucosa (*Arrow*) H&E, X 62,50. **b** Trachea of IBV/TU inoculated chick – moderate hyerplasia of surface epithelium and loss of cilia (*Arrow1*) and moderate lympho- plasmocytic infiltration of lamina propria (*Arrow2*), H&E, X250

In the trachea, most detected lesions in all infected groups were characterized by mucosal thickening, hyperplasia of the surface epithelium, loss of cilia, and mononuclear inflammatory cell infiltrate (lymphocytes, plasmocytes, and macrophage) of lamina propria (Figs. 2a and b). Mean total score of severity was 3,7; 4,7 and 6,7 in birds infected by IBV/MN; IBV/RA and IBV/14/TU strains respectively. This indicates that IBV/TU strain seems to have a higher degree of pathogenicity than the other tested strains (Table 4).

**Table 4:** Microscopic lesion scores in trachea of 5 day-old SPF chickens inoculated with field IBV strains IBV/MN, IBV/RA and IBV/TU*IBV* infectious bronchitis virus

	Lesions		Mean lesion scores in IBV infected groups of birds		
			IBV/MN	IBV/RA	IBV/TU
Trachea	Epithelium	Thickening	1	1,3	2
		Hyperplasia (1-4 +)	1	1,3	2,6
		Loss of cilia	0	0	0,6
		Degenerescence and necrosis of epithelial cells	0	0	0
		Inflammatory exudate (degenerate epithelial cells, Lc, heterophyles) on the surface	0	0,3	0,6
	Lamina propria	Oedema,	0	0,6	1
		Diffuse infiltration by mononuclear inflammatory cells (Lc, Plc, Mph)	1,3	1,3	1,3
		Mean total Score of severity	3.666	4.666	7,666

*IBV* infectious bronchitis virus

*SPF* Specific pathogen free

*IBV/TU* infectious bronchitis virus sampled from Abda-Doukkala region

*IBV/RA* infectious bronchitis virus sampled from Rabat-Sale-Azzemour Zaire region

*IBV/MN* infectious bronchitis virus sampled from Meknes Tafilalet region

**Fig. 3:**
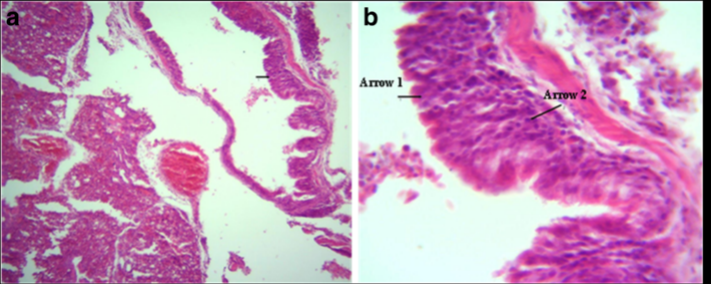
**a** Lung of IBV/MN inoculated chick – thickening of the surface mucosa (*Arrow*) of a secondary bronchus, H&E, X 62,50. **b** Lung of IBV/MN inoculated chick – mild hyerplasia of surface epithelium (*Arrow 1*) and severe heterophilic and lympho-plasmocytic infiltration of Lamina propria (*Arrow 2*) and paeribronchial space of a secondary bronchus, H&E, X250

In the lungs, lesions in all infected groups were mainly confined to the primary and secondary bronchi and included epithelial hyperplasia, loss of cilia, and heterophils and mononuclear inflammatory cell infiltrate (lymphocytes, plasmocytes and macrophages) of the lamina propria and peribronchial space (Fig. 3a and b). Other changes were located in the atria and parabronchi and consisted of hypertrophy and/or hyperplasia of pneumocytes. The score of these changes were from mild to moderate degree of severity and there was no big differences in the mean total score of severity among tissues infected with the 3 tested IBV strains (7,3; 8,3 and 6), thus indicating no differences in the pathogenicity of the three strains for the lung (Table 5). However, no kidney lesions were observed in chicks infected with the three tested strains. Tracheas and lungs of control chicks samples throughout the experiment, showed normal histological features.

**Table 5:** Microscopic lesion scores in lungs of 5 day-old SPF chickens inoculated with field IBV strains IBV/MN, IBV/RA and IBV/TUIBV: infectious bronchitis virus

	Lésions		Mean lesion scores in IBV infected groups of birds		
			IBV/MN	IBV/RA	IBV/TU
Lung	Primary & secondary Bronchi	Epithelial hyperplasia	2	1,7	1
		Loss of cilia	1	1	0,7
		peribronchial infiltration by mononuclear inflammatory cells	2	1,3	0,3
		Infiltration of lamina propria by heterophils & mononuclear inflammatory cells	2	1,3	0,3
	Atria & parabronchi	Hypertrophy of pneumocytes	0	0,7	1,3
		Epithelial hyperplasia	0	0,7	1,3
	Inter-capillarysepta	hyperemia	1	1	1
		Mean of total Score of severity	7.333	7.666	6

*IBV* infectious bronchitis virus

*SPF* Specific pathogen free

*IBV/TU* infectious bronchitis virus sampled from Abda-Doukkala region

*IBV/RA* infectious bronchitis virus sampled from Rabat-Sale-Azzemour Zaire region

*IBV/MN* infectious bronchitis virus sampled from Meknes Tafilalet region

### Ciliostasis: tracheal ciliary activity

Ciliary activity was observed in all tracheas of infected birds at 5dpi. But the severity differed between the three strains tested; the ciliostasi caused by IBV/MN and IBV/RA strains was 25 % and 50 % for IBV/TU strain, compared to the control group (100 %). However, a partial recovery of ciliary activity in infected birds was observed at 12dpi.

### Viral re- isolation

**Fig. 4:**
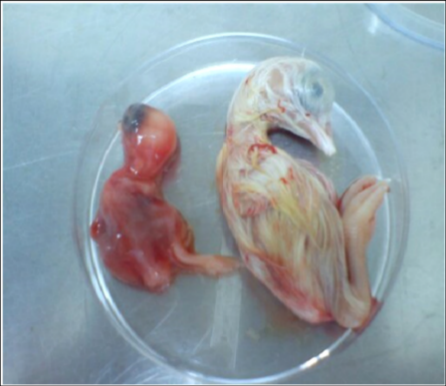
Dwarfism and hemorrhage of embryos after re-isolation of the virus from experimentally infected chicks (in the left) in comparison with the control (in the right)

Results related to IBV distribution was checked in all tissues samples collected at 5dpi of all infected chicks. The virus was found in the trachea, lung of all strains, but in the kidney of one strain (IBV/MN) with low viral load. The effect of the virus after one passage in SPF 10 old embryonated eggs showed dwarfism and hemorrhagic spots in all over the body of the embryo, which are considered pathognomonic signs of IBV (Fig. 4).

The virus was re-isolated from all the sampled tissues with a high viral load in the trachea and lungs. No IB virus was re-isolated from the control group. Real time RT-PCR results obtained from the tissues of infected chicks at 5 dpi was closely correlating with those obtains from allantoic liquid of inoculated eggs (Table 6).

**Table 6:** Viral replication detected 5 days post infection in the respiratory and nephritic tract of experimentally infected chicks*SPF* Specific pathogen free

Real time RT-PCR (Ct) for						
Viral strains	Organs samples			Fluid SPF eggs		
	Trachea	Lung	Kidney	Trachea	Lung	Kidney
IBV/TU	27.05	32.49	0	13.13	20.30	0
IBV RA	28.73	25.43	0	14.79	14	0
IBV/MN	30.21	27.3	30.74	13	21.41	30.74
Control	0	0	0	0	0	0

*SPF* Specific pathogen free

*rRT- PCR* real time reverse transcriptase polymerase chain reaction

*Ct* Cycle threshold

*IBV* infectious bronchitis virus

*IBV/TU* infectious bronchitis virus sampled from Abda-Doukkala region

*IBV/RA* infectious bronchitis virus sampled from Rabat-Sale-Azzemour Zaire region

*IBV/MN* infectious bronchitis virus sampled from Meknes Tafilalet region

### Serological response

Antibody titers against Infectious Bronchitis (Anti-IBV) in the serum collected from chickens of different groups on days 5 and 14 dpi was measured by ELISA test. In the all infected group, serum samples were negative at day 0 and 5 dpi but antibodies titers was increased at 14dpi. Serum of the control chicks was free from specific antibodies against IBV on the entire dpi.

## Discussion

In the present work, we have studied the pathogenesis of IBV Italy 02 genotype which was recently detected for the first time in Morocco and also in Africa [15]. No studies on the Italy02 genotype were reported in neighboring Maghreb or other African countries. The previous study in Morocco focused the mapping serotypes circulating in poultry flocks [15], but no information is available about the pathogenicity and the tissue tropism of this new genotype (Italy02) and protective efficacy of implemented vaccines. To do that, experimental infection in one day-old SPF chickens was used to evaluate pathogenicity and tissue tropism of three Moroccan Italy02 strains (coded, IBV/MN, IBV/TU and IBV/RA).

Results showed that all the three tested strains of IBV, were capable to induce respiratory signs at 48 h pi. Infected chicken showed the various signs of depression, sneezing, difficulty in breathing, nasal discharge, cough, pulmonary and tracheal rales. Developed symptoms became severe at 5 to 7 dpi, and persisted in some chicks until 12dpi. The birds infected by IBV/MN and IBV/TU appeared lethargic, reluctant to move compared to those infected by IBV/RA. No clinical nephritis or mortalities were recorded during the course of experiment in all groups. The clinical signs and gross lesions of this genotype are in accordance with the findings that have been described previously for other serotype of IBV [18], and were in agreement with the clinical signs mentioned by Purcell and McFerran [19]. Regarding to histopathological examination of 3 sacrificed infected birds at 5dpi. The three IBV strains studied have the same severity of lesions in lung with no differences in the pathogenicity. But the IBV/TU strain seems to have a higher degree of pathogenicity in the trachea than others tow tested strains. Clinical signs score and tracheal lesions developed in all infected birds confirm the ability of this new Moroccan genotype, to induce respiratory disease and is therefore regarded as having respiratory tropism. Whereas, Dolz et al, demonstrated that the Spanish Italy02 serotype capable to cause severe respiratory and renal damage [20]. The pathogenicity and clinical sing score obtained with the three strains Italy02 genotype were in accordance and common with those reported for most other serotype of IBV in different countries [21–24].

Results of virus re-isolation confirm IBV distribution in all tissues examined at 5dpi, with the three tested strains. Histopathological and gross lesions showed that the trachea is the primary site of lesions associated with IBV replication as generally reported for the IBV virus [25].

In this report, ELISA test used to evaluate levels of the Ab to IBV in all infected groups at 5 and 14dpi. ELISA method detected moderate levels of the Ab at 14dpi. This result confirms that infected chickens seroconverted against IB, after manifestation of specific clinical sings. Indeed, Ghadakchi et al. showed that ELISA could be reliable, repeatable, and sensitive for detecting IB antibodies [26].

Results obtained with Moroccan Italy02 genotype demonstrated that the three strains emerging in Moroccan poultry farms have a wide distribution for respiratory system, without kidney damage and without causing mortality.

## Conclusion

The present study has revealed that the chickens infected with the three tested strains showed the specific respiratory signs with high clinical signs. These infected chicks were sero-converted at 14 dpi and confirmed the spread of the inoculated virus. In addition, the macroscopic lesions and histopathological examination revealed specific lesions of trachea and lung, without renal manifestations. The histopathology of respiratory organs demonstrated a high lesional score of IBV/TU strain which correlate with the clinical score of 140. Moreover, virus re-isolation was performed successfully for all the three tested strains. Results obtained with real time PCR in organs sampled from infected birds (trachea, lung and kidney) and from fluid allantoic in the first passage in SPF eggs, showed a significant abundance of the virus in the respiratory tract. In other words, the findings of molecular analysis of virus re-isolation are strongly correlated with the histopathological tests.

Finally, this current report justify that Italy 02 genotype isolated from different regions of Morocco is capable to induce a severe respiratory disease with a wide distribution of respiratory system. However, this genotype do not cause any kidney damage and without causing mortality.

## Methods

### Viral strains

Three IBV field strains (coded, IBV/MN, IBV/RA and IBV/TU), were isolated from trachea, lungs and kidney of broiler chickens suffering from the specific clinical signs of IB, and were previously characterized to belong to Italy02 serotype at laboratory of Biopharma between 2010- 2014 [15]. Phylogenetically, the three strains of Italy02 genotype branched and clustered with Spanish genotypes, are very closely related to Italy 497/02-1 (98.9 %); Spain/05/866 (98.9 %); and Spain/04/221 (97.4 %). The sequences of the three strains have a S1 gene nucleotide identity ranged between 96.9 % and 98.7 % when compared to each other.

These IBV strains were propagated by inoculation in 9- to 11-day-old SPF egg as described by Owen et *al* [27]. For virus titration 6-fold dilutions were inoculated into the allantoic cavity of six SPF chickens embryos, and incubation during six days at temperature of 37 °C +/- 2 °C and relative humidity of 75 % +/- 10 %. The titer is calculated following the Reed and Muench method [28].

### Chickens

A total of 40 one day old SPF chickens, used for experimental infection were provided from the SPF chickens flock unit of Biopharma Laboratory, Rabat, Morocco.

### Experimental infections

In order to evaluate the pathogenesis of three Moroccan viral strains, an experimental infection study was performed in SPF chickens obtained from SPF chicken flock Unit of Biopharma, Rabat, Morocco. For this purpose, 40 one-day-old SPF chickens were housed in separate isolators with negative-pressure, the bright and thermal program was the same for all birds, and water and food were provided at will. Chickens were randomly divided into four groups of 10 birds (30 chicks in the experimental and ten chicks in the control group). The infection trials were approved by the local ethics committee of Biopharma (Biopharma Ethics and Scientific Committee) directed by its veterinarian in charge*,* with requirement of formal ethics approval referenced as **N**° **412**/**SB**/**2015**, and were carried out following strict animal welfare guidelines.

Using a micropipette, chickens in the three groups were oculo-nasally administrated with 50 μl of inoculum containing 10^3.5^ (EID50/ml) of virus strain, and control group was inoculated with phosphate-buffered saline (1XPBS). The specific clinical signs of IB were recorded at a rate of 2 times daily by the same observer during 14 days. Specific clinical signs of IB recorded by the same observer have included gasping, coughing, sneezing, depression, rales, and ruffled feather. Three chickens from each group were killed at 5dpi; one control animals and their tissues of trachea, lung and kidney were examined for gross lesions, and were collected for histopathology examination. Serum samples were tested for the presence of antibodies against IB using the ELISA test. Also the tracheal rings from each bird were obtained and placed in cell culture medium (D-MEM supplemented with 10 % of foetal calf serum) to evaluate tracheal ciliary activity at 5 and 12 dpi. Treatment organs of lung, trachea and kidney by the antibiotics and frozen at -80 °C were used for virus re-isolation and real time RT-PCR detection. At 14 dpi, all the birds were euthanized and necropsied for gross lesions examination before their elimination.

### Histopathological examination

Sacrificed birds at 5 days of age were necropsied and gross lesions were recorded. Tissue samples from the trachea and lungs were collected and fixed in 10 % neutral-buffered formalin. They were processed for histopathological examination according to standard methods. They were dehydrated and embedded in paraffin wax. Five μm thick sections were stained with hematoxylin and eosin and examined under light microscope. Microscopic lesions in respiratory tissues were recorded and scored as described previously [17], from 0, 1, 2 and 3 (no, mild, moderate and severe, respectively). A mean of total severity index was then attributed to each IBV strains based on scores from 3 birds.

### Ciliostasis: tracheal ciliary activity

Several parts of tracheal rings were prepared from each infected bird at 5dpi that coincides with the pic of the clinical signs. At 12 dpi, the ciliary activity was also evaluated from all the infected birds that have recovered. The prepared tracheal rings were immersed immediately in cell culture medium (D-MEM supplemented with 10 % of foetal calf serum), were microscopically analyzed for estimating the ciliary movement in tracheal infected and in tracheal control, and were scored on a scale from 0 (100 % activity) to 4 (no activity) [29]. The mean score of each strain was then compared to evaluate the respiratory pathogenicity.

### Viral re-isolation

All individual samples of trachea, lung and kidney collected at 5 dpi from IBV-infected chickens, were immediately suspended in phosphate-buffered saline (1XPBS), treated by Gentamycin antibiotics (50 μg/g) and clarified by centrifugation at 2500 × g at 4 °C for 25 min. The homogenates samples were used and processed for IBV re-isolation as previously described [30]. It’s was performed by inoculation in allontoic sac route of 9–11 day old eggs, with 100 μl of homogenates and incubation during 6 days (using five eggs by sample) at temperature of 37 °C +/- 2 °C and relative humidity of 75 % +/- 10 %. The embryos were examined for the presence of dwarfism and hemorrhagic traces (on the whole body of the embryo). The RT-PCR detection of viral RNA was performed in parallel with the homogenates tissues and the clarified allontoic fluid.

### Extraction of viral RNA

Viral RNA was extracted from 200 μl of both organs samples and allantoic fluid (AF) using the Kits Macherey-Nagel according to the manufacturer’s instructions. Each RNA fraction was eluted in 50 μl of RNase-free water, and subjected to reverse transcriptase RT-PCR amplification.

### Real time RT-PCR

A one step Real time RT-PCR was carried out using Invitrogen kit (SuperScript® III Platinum, Life Technologies, USA). The synthesis of the cDNA first strand was performed using 5 μl total viral RNA primed with a universal pair of primers a) downstream primer, AIBV-fr, targeting N gene nucleotide positions 811–832 (5′-ATGCTCAACCTTGTCCCTAGCA-3′); and b) upstream primer, AIBV-as, targeting N gene nucleotide positions 921–941 (5′-TCAA-ACTGCGGATCA-TCACGT-3′), and the probe TaqMan targeting N gene nucleotide positions 848-875 (5′FAM-TTGGAAGTAGAGTGACGCCCAAACTTCA-3′Tamra) [31].

The amplifications reactions (PCRs) were performed on a Smart Cycler. The following mix of each reaction was contained: 12.5 μl 2 × RT- PCR buffer mix, 0.5 μl MgSO4 (50 mM), 0.5 μl Rox (25 mM), 4.75 μl nuclease free water, 0.5 μl M-MULV reverse transcriptase enzyme (200U), 0.5 μl primers to a final concentration of 10 μM, 0.25 μl probe to a final concentration of 10 μM and 5 μl RNA template. The reaction was carried out in StepOneTM Plus real-time PCR system (Smart cycler Cepheid, USA) at 50 °C for 15 min, 95 °C for 5 min, and 40 cycles of 95 °C for 15 s and 60 °C for 45 s. All reactions amplifications were recorded, analyzed, and the threshold cycle (Ct) determined with the StepOne software (Smart Cycler).

### Serological response

An enzyme-linked immunosorbent assay (ELISA) commercial kit (FlockChek (IDEXX Laboratories, Inc., Westbrook, ME, USA), was carried out on serum samples collected from experimentally infected birds for antibodies detection at 5 and 14dpi. The test was performed according to the manufacturer’s instructions. Moreover, serology on SPF chicks of one day from the same rearing was also performed to ensure the negative status anti-IBV chicks antibodies used.

## Abbreviations

Ct, cycle threshold; dpi, day post-infection; IB, infectious bronchitis; IBV/MN, infectious bronchitis virus sampled from Meknes Tafilalet region; IBV/RA, infectious bronchitis virus sampled from Rabat-Sale-Azzemour Zaire region; IBV/TU, infectious bronchitis virus sampled from Abda-Doukkala region; IBV, infectious bronchitis virus; M, membrane; N, nucleocapsid; Pi, post-infection; rRT- PCR, real time reverse transcriptase polymerase chain reaction; S, spike; SPF, specific pathogen free.
